# Association of the C-reactive protein–triglyceride–glucose index with the risk of incident cardiovascular disease in rectal cancer survivors

**DOI:** 10.3389/fcvm.2026.1880389

**Published:** 2026-07-29

**Authors:** Qiming Zhao, Chen Zhang, Wenhui Jiang, Wei Liu, Fangmin Shi, Chengnan Liu, Minglian Ouyang, You Guo

**Affiliations:** 1 Medical Big Data and Bioinformatics Research Center, First Affiliated Hospital of Gannan Medical University, Ganzhou, China; 2 School of Public Health and Health Management, Gannan Medical University, Ganzhou, China; 3 Ganzhou Key Laboratory of Medical Big Data, Ganzhou, China; 4 Gannan Subcenter for Medical Genomics, National Genomics Data Center, Ganzhou, China; 5 First School of Clinical Medicine, Gannan Medical University, Ganzhou, China

**Keywords:** c-reactive protein-triglyceride-glucose index, incident cardiovascular disease, inflammation, insulin resistance, rectal cancer, risk stratification

## Abstract

**Background and objective:**

Rectal cancer survivors face an elevated cardiovascular disease (CVD) burden shaped by tumor- and treatment-related pathophysiology beyond conventional risk factors. We evaluated the association between the C-reactive protein–triglyceride–glucose index (CTI)—a composite inflammatory–metabolic indicator—and incident CVD in this population.

**Methods:**

This retrospective cohort study enrolled 1,160 rectal cancer survivors from a Chinese tertiary hospital as the discovery cohort and 1,752 from the UK Biobank as an independent validation cohort. Incident CVD was the primary outcome. Multivariable logistic regression, restricted cubic splines, and model performance metrics were used to assess the association, dose–response relationship, and incremental predictive value of CTI.

**Results:**

After full multivariable adjustment, each one-SD increment in CTI was associated with an 18% higher risk of incident CVD (OR = 1.18, 95% CI: 1.02–1.36, *p* = 0.025), with an approximately linear dose–response relationship (p for non-linearity = 0.448) and excess risk concentrated in the highest quartile (Q4 vs. Q1: OR = 1.60, 95% CI: 1.07–2.42, *p* = 0.024). Adding CTI improved discrimination (AUC = 0.718) beyond the CRP-based model (AUC = 0.713, *p* = 0.050) and the TyG-based model (AUC = 0.715, *p* = 0.042), and significantly improved risk reclassification (categorical NRI = 0.052, 95% CI: 0.018–0.086, *p* = 0.005). The association was stronger in patients aged <65 years (OR = 1.63; *p* for interaction = 0.007) and those without baseline hypertension (OR = 1.33; *p* for interaction = 0.034). In the UK Biobank cohort, the CTI–CVD association attenuated progressively and became non-significant after full adjustment (OR = 1.08, 95% CI: 0.95–1.22, *p* = 0.261; AUC = 0.649).

**Conclusion:**

CTI was independently associated with incident CVD in rectal cancer survivors and improved risk reclassification beyond CRP or TyG alone, with effects concentrated in younger and non-hypertensive patients. Its incremental value attenuated in a community-based population, suggesting that CTI is most informative in oncology settings with high inflammatory–metabolic burden.

## 1 Introduction

Advances in multimodal treatment strategies have substantially improved long-term survival among rectal cancer patients, shifting clinical priorities from short-term tumor control toward the prevention and management of late complications (1, 2). Among these, non-neoplastic conditions—particularly cardiovascular disease (CVD)—have emerged as important determinants of long-term prognosis in rectal cancer survivors (3). Population-based studies and registry data consistently demonstrate that cancer survivors experience a higher risk of cardiovascular events than the general population, with CVD now representing one of the leading contributors to morbidity and mortality in this group (4–6). These findings underscore the clinical importance of systematic cardiovascular risk assessment during oncological follow-up and long-term management. Consistently, recent cardio-oncology guidelines emphasize the importance of comprehensive cardiovascular risk evaluation and long-term surveillance in cancer survivors to identify individuals at increased risk of cardiovascular complications (7, 8).

The cardiovascular risk profile of rectal cancer survivors is shaped by the interplay between tumor-related biology and traditional cardiovascular risk determinants. Anticancer treatments—including surgery, radiotherapy, and chemotherapy—may cause cardiovascular injury through systemic inflammatory activation, endothelial dysfunction, and direct cardiotoxicity (9–11). Although conventional risk factors such as hypertension and diabetes remain clinically relevant, they do not fully account for the excess cardiovascular burden observed in this population, implying that tumor- and treatment-related mechanisms contribute to residual risk (12, 13).

Systemic inflammation and insulin resistance are two mechanistically interconnected pathways through which cardiovascular injury may occur. C-reactive protein (CRP), an established marker of systemic inflammation, is independently associated with cardiovascular events across diverse populations (14). The triglyceride–glucose (TyG) index, a validated surrogate of insulin resistance, has demonstrated predictive value for atherosclerosis progression and adverse cardiovascular outcomes (15–17). However, inflammatory and metabolic biomarkers have largely been evaluated in isolation. In rectal cancer survivors exposed to concurrent tumor-associated and treatment-related physiological disturbances (18), single biomarkers may fail to capture the multidimensional inflammatory–metabolic dysregulation underlying cardiovascular vulnerability.

Against this background, composite inflammatory–metabolic indices have attracted growing interest as more integrative tools for cardiovascular risk stratification in cancer survivors. The C-reactive protein–triglyceride–glucose index (CTI), derived by multiplying CRP by the TyG index, simultaneously captures systemic inflammatory activation and insulin resistance within a single calculable metric, and has been proposed as a practical tool for cardiovascular risk assessment (19). Prospective evidence further links elevated CTI to coronary artery calcium progression (20), and adverse cardiometabolic outcomes (21), supporting its broader cardiovascular relevance. Nevertheless, direct evidence regarding CTI in cardio-oncology settings—particularly among rectal cancer survivors who carry a distinct inflammatory–metabolic burden shaped by tumor biology and treatment exposure—remains absent.

Accordingly, this study evaluated the association between CTI and incident CVD in a real-world cohort of Chinese rectal cancer patients, with external validation in rectal cancer participants from the UK Biobank. We investigated whether this readily calculable composite indicator could provide incremental information for cardiovascular risk stratification in cardio-oncology practice.

## 2 Materials and methods

### 2.1 Study design, data sources, and ethical approval

This retrospective cohort study combined a primary analysis cohort with a supplementary external validation cohort. Data for the primary analysis cohort were extracted from the First Affiliated Hospital of Gannan Medical University. Data for external validation were obtained from the UK Biobank.

The study protocol was approved by the Ethics Review Committee of the First Affiliated Hospital of Gannan Medical University, and the requirement for informed consent was waived due to the retrospective nature of the data analysis. Ethical approval for the UK Biobank was granted by the North West Multi-centre Research Ethics Committee (approval number: 299116, available at: https://biobank.ndph.ox.ac.uk/). All participants provided written informed consent. Our analyses were conducted under UK Biobank application number 532253 (the official approval documentation is provided as Supplementary Document 1) and received appropriate ethical approval. This study adhered strictly to the ethical principles of the Declaration of Helsinki.

### 2.2 Study population

#### 2.2.1 Primary analysis cohort (Chinese cohort)

A total of 1,160 patients with rectal cancer were identified from the First Affiliated Hospital of Gannan Medical University between January 1, 2010, and March 31, 2024. Rectal cancer diagnosis was defined using the International Classification of Diseases, Tenth Revision (ICD-10) codes C19-C20.

Inclusion criteria were: (1) age ≥18 years at diagnosis; (2) at least two hospital admission records; (3) complete baseline demographic and clinical data, along with laboratory data requisite for calculating the CTI; and (4) available follow-up information to determine the primary outcome.

Exclusion criteria were: (1) missing key variables required for CTI calculation (CRP, triglycerides, or glucose); (2) presence of other severe or life-threatening diseases; (3) loss to follow-up, transfer to another hospital, or discharge against medical advice during diagnosis or treatment, precluding outcome determination; and (4) documented CVD prior to rectal cancer diagnosis, to ensure the study outcome was an incident event and maintain temporal plausibility.

#### 2.2.2 Supplementary validation cohort (UK biobank)

The supplementary validation cohort was derived from the UK Biobank database, comprising 1,752 patients with rectal cancer between January 2006 and August 2025. The same ICD-10 codes (C19–C20) were used to identify patients with rectal cancer, and the same inclusion and exclusion criteria as those applied to the primary analysis cohort were used to ensure comparability of the study populations. This cohort served as an independent sample for external validation of the model and for attenuation effect analysis.

**Figure 1:**
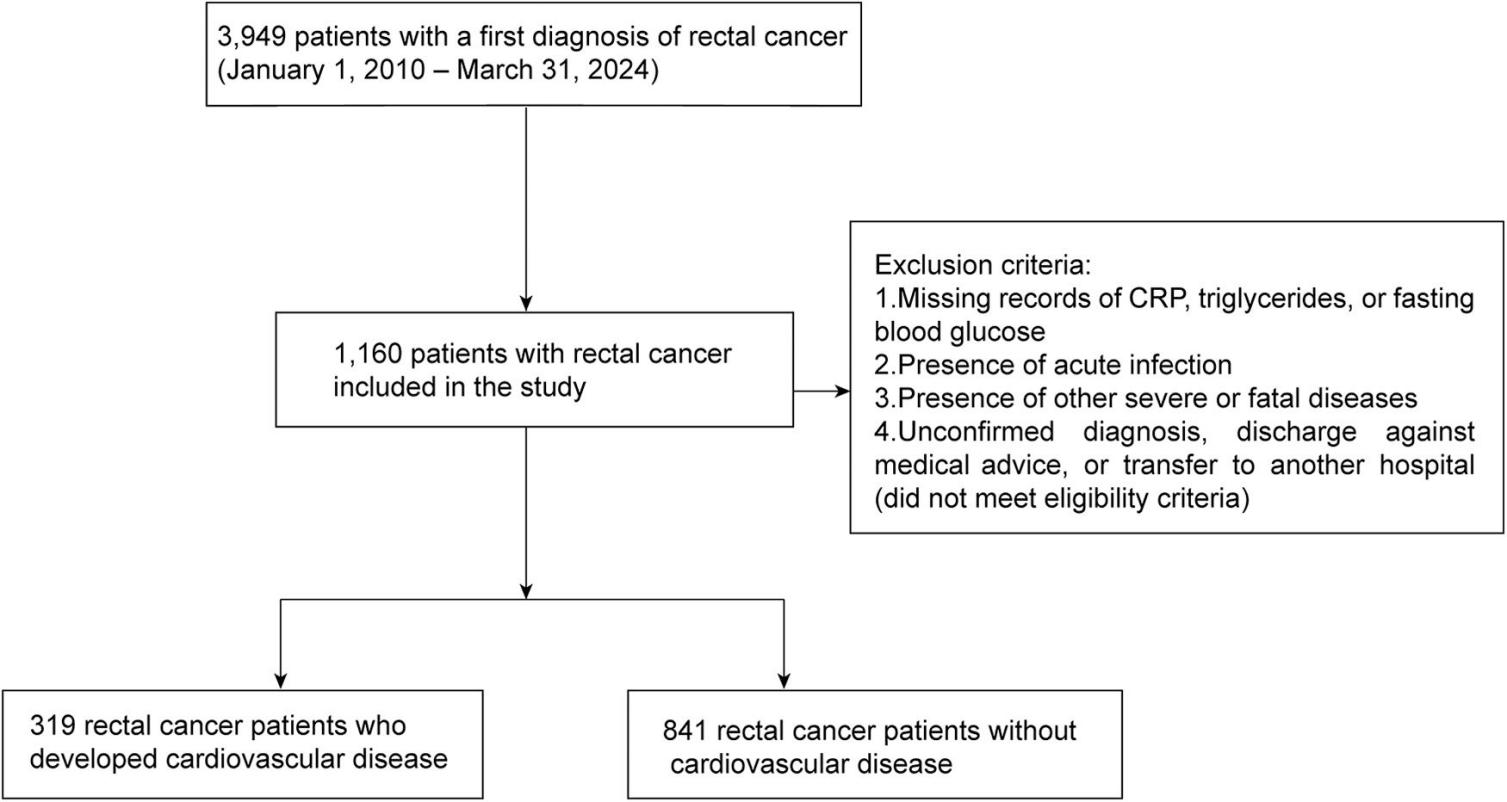
Study flow diagram for patient selection in the primary Chinese cohort. Flow chart illustrating the selection process of rectal cancer patients from the First Affiliated Hospital of Gannan Medical University between January 1, 2010, and March 31, 2024. A total of 3,949 patients with an initial diagnosis of rectal cancer were identified. After applying the predefined exclusion criteria, 1,160 patients were enrolled in the final analysis cohort. Among these, 319 patients developed incident cardiovascular disease (CVD) during follow-up, while 841 patients remained free of CVD. CRP, C-reactive protein; CVD, cardiovascular disease; CTI, C-reactive protein-triglyceride-glucose index; ICD-10, International Classification of Diseases, Tenth Revision.

Detailed screening processes for the study subjects are shown in Figure 1. The screening process for the UK Biobank validation cohort is shown in Supplementary Figure S1.

### 2.3 Exposure, outcome, and covariates

#### 2.3.1 Exposure variable: CTI

The primary exposure variable in this study was the baseline CTI. All laboratory indicators were derived from fasting venous blood samples collected during the baseline hospital admission following the initial diagnosis of rectal cancer.

CTI was calculated using the following formula:

$$\mathrm{CTI}=0.412\times \ln(\mathrm{CRP}[\mathrm{mg}/L])+\frac{\ln(\mathrm{TG}[\mathrm{mg}/\mathrm{dL}]\times \mathrm{FPG}[\mathrm{mg}/\mathrm{dL}])}{2}$$

where CRP represents C-reactive protein, TG represents triglycerides, and FPG represents fasting plasma glucose (12).

#### 2.3.2 Outcome variable: incident CVD

The primary outcome of this study was incident CVD in patients with rectal cancer, defined as a composite endpoint. Specifically, we assessed whether patients receiving rectal cancer-related treatment during hospitalization developed one or more new-onset cardiovascular events during follow-up from 3 months to 6 years post-discharge. The 3-month lag period was implemented to exclude perioperative or acute treatment-related cardiovascular complications.

The diagnosis of incident CVD was defined according to chapter IX (Diseases of the circulatory system, I00–I99) of the ICD-10 codes, encompassing the following nine disease categories: coronary artery disease, heart failure, arrhythmias, pulmonary hypertension, valvular heart disease, pericardial disease, peripheral vascular disease, stroke, and thrombotic disease. Patients who developed any of these CVDs during follow-up were identified as having experienced an incident CVD event (18).

#### 2.3.3 Covariates

Based on previous literature and clinical relevance, the following variables were included as potential confounders for statistical adjustment. Demographic characteristics included age and sex; anthropometric measurements included body mass index (BMI), systolic blood pressure (SBP), and diastolic blood pressure (DBP); clinical history included history of hypertension and diabetes mellitus; treatment-related variables included history of surgery and history of chemotherapy. Laboratory indicators included white blood cell count, neutrophil count, lymphocyte count, platelet count, serum albumin, fibrinogen, estimated glomerular filtration rate (eGFR, calculated using the CKD-EPI equation), low-density lipoprotein cholesterol (LDL-C), high-density lipoprotein cholesterol (HDL-C), and eosinophil count.

### 2.4 Handling of missing data

To mitigate potential bias introduced by missing data, multiple imputation was employed to handle missing values. Specifically, the Multiple Imputation by Chained Equations (MICE) method was used to generate five imputed datasets. All subsequent statistical analyses were performed separately on each imputed dataset, and the results were combined according to Rubin's rules to obtain pooled parameter estimates and standard errors (22). The proportion of missing values for all key variables, including CTI components, outcome, and covariates, is presented in Supplementary Table S1. Core variables had no missing values or minimal missingness, while missingness in secondary covariates ranged from 0.1% to 21.6%.

### 2.5 Statistical analysis

Continuous variables are summarized as mean ± standard deviation or median (interquartile range) according to their distributional properties; categorical variables are reported as frequencies with percentages. Between-group comparisons were performed using one-way ANOVA, the Kruskal–Wallis test, or the *χ*^2^ test, as appropriate.

The association between CTI and incident CVD was assessed using multivariable logistic regression, with results reported as odds ratios (OR) and 95% confidence intervals (CI). Three progressively adjusted models were fitted: Model 1 adjusted for age, sex, and BMI; Model 2 additionally adjusted for history of hypertension, history of diabetes, and eGFR; and Model 3—the fully adjusted model—further adjusted for history of surgery and history of chemotherapy. Within the fully adjusted model, restricted cubic splines (RCS) with four knots were used to characterize the dose–response relationship between CTI and incident CVD and to test for departure from linearity. As a secondary exploratory analysis, incident CVD was further classified into three mechanistic categories—cardiac dysfunction–related events (heart failure, arrhythmia, pericardial disease), thromboembolic and pulmonary vascular events (thrombotic disease, pulmonary hypertension), and atherosclerotic vascular events (coronary artery disease, stroke, peripheral vascular disease)—and each category was modeled using the same covariate set as the primary analysis.

Least Absolute Shrinkage and Selection Operator (LASSO) regression was applied to screen candidate predictors, and the final risk-prediction model combined LASSO-selected variables with those judged to be clinically relevant. The incremental value of CTI was assessed by comparing model fit (AIC and −2 log-likelihood) and discrimination (AUC) across three single-marker models: a CRP model, a TyG model, and a CTI model, each incorporating the respective marker alongside the same base covariates. AUC differences between models were evaluated using DeLong's test. Reclassification was quantified using the categorical Net Reclassification Improvement (NRI) and Integrated Discrimination Improvement (IDI); predicted risk was categorized as low (<10%), intermediate (10%–20%), or high (>20%).

Four modeling algorithms—GLM, KNN, RF, and SVM—were compared for incident CVD prediction using fivefold cross-validation in the primary cohort, and the optimal algorithm was selected as the final model based on overall cross-validation performance. The final model was developed on the primary cohort and internally validated using 1,000-iteration bootstrap resampling; external validation was subsequently performed in the UK Biobank cohort.

SHAP (SHapley Additive exPlanations) analysis was used to visualize the relative contribution of each predictor within the final model, and a nomogram was constructed to facilitate individualized risk estimation. Subgroup analyses were stratified by age, history of hypertension, and history of diabetes; multiplicative interaction terms were tested to evaluate potential effect modification. Sensitivity analyses were conducted by sequentially excluding predefined subpopulations, including a separate analysis excluding participants with baseline CRP > 10 mg/L, under the fully adjusted model. In the UK Biobank cohort, attenuation analyses were performed by sequentially adjusting for demographics (age, sex), lifestyle factors (BMI, smoking, alcohol consumption), comorbidities (hypertension, diabetes), renal function (eGFR), and treatment history (surgery, chemotherapy).

All analyses were performed in R (version 4.5.1). All tests were two-sided, with *p* < 0.05 considered statistically significant.

## 3 Results

### 3.1 Baseline characteristics of the study population

The 1,160 rectal cancer survivors were stratified into quartiles (Q1–Q4, *n* = 290 each) based on baseline CTI levels. The baseline characteristics of the study population are detailed in Table 1.

**Table 1:** Baseline characteristics of the study population according to CTI quartiles.

Variable	Q1	Q2	Q3	Q4	Total	*P*
	(*N* = 290)	(*N* = 290)	(*N* = 290)	(*N* = 290)	(*N* = 1,160)	
**Gender**						0.352
Male	181 (62.4%)	186 (64.1%)	198 (68.3%)	179 (61.7%)	744 (64.1%)	
Female	109 (37.6%)	104 (35.9%)	92 (31.7%)	111 (38.3%)	416 (35.9%)	
**Age**						0.016
Mean ± SD	60.37 ± 11.92	62.30 ± 11.58	63.41 ± 12.29	63.49 ± 12.65	62.39 ± 12.17	
Median (IQR)	62.00 (52.00, 69.00)	62.00 (55.00, 70.00)	64.00 (56.00, 71.00)	64.00 (54.25, 72.00)	63.00 (54.00, 71.00)	
**CTI**						<0.001
Mean ± SD	4.24 ± 0.36	4.99 ± 0.17	5.59 ± 0.20	6.37 ± 0.34	5.30 ± 0.83	
Median (IQR)	4.33 (4.04, 4.52)	4.99 (4.84, 5.13)	5.59 (5.41, 5.79)	6.33 (6.09, 6.57)	5.26 (4.67, 5.94)	
**CRP**						<0.001
Mean ± SD	0.84 ± 0.64	3.54 ± 2.78	14.54 ± 12.04	79.18 ± 63.17	24.52 ± 45.34	
Median (IQR)	0.70 (0.41, 1.10)	2.75 (1.81, 4.37)	12.00 (6.50, 18.52)	61.27 (43.86, 95.31)	5.00 (1.33, 25.72)	
**TyG**						<0.001
Mean ± SD	8.17 ± 0.42	8.43 ± 0.46	8.54 ± 0.56	8.69 ± 0.58	8.46 ± 0.54	
Median (IQR)	8.17 (7.89, 8.45)	8.41 (8.11, 8.72)	8.47 (8.14, 8.92)	8.59 (8.28, 9.06)	8.40 (8.09, 8.79)	
Albumin						<0.001
Mean ± SD	40.61 ± 4.25	40.11 ± 4.33	38.61 ± 5.34	37.39 ± 6.48	39.18 ± 5.33	
Median (IQR)	40.05 (37.85, 43.40)	40.25 (37.45, 42.90)	39.10 (35.90, 42.50)	38.10 (33.60, 41.70)	39.50 (36.30, 42.70)	
Eosinophil Count						<0.001
Mean ± SD	0.16 ± 0.15	0.19 ± 0.26	0.17 ± 0.21	0.15 ± 0.38	0.17 ± 0.26	
Median (IQR)	0.12 (0.07, 0.21)	0.14 (0.07, 0.22)	0.12 (0.06, 0.20)	0.10 (0.04, 0.16)	0.12 (0.06, 0.20)	
Basophil Count						0.039
Mean ± SD	0.03 ± 0.02	0.07 ± 0.66	0.03 ± 0.02	0.03 ± 0.02	0.04 ± 0.33	
Median (IQR)	0.02 (0.01, 0.03)	0.03 (0.01, 0.04)	0.03 (0.01, 0.04)	0.02 (0.01, 0.04)	0.02 (0.01, 0.04)	
Lymphocyte Count						0.001
Mean ± SD	1.53 ± 0.56	1.67 ± 0.95	1.54 ± 0.60	1.44 ± 0.71	1.54 ± 0.73	
Median (IQR)	1.48 (1.13, 1.89)	1.53 (1.23, 2.00)	1.54 (1.12, 1.92)	1.38 (0.92, 1.89)	1.48 (1.11, 1.90)	
Neutrophil Count						<0.001
Mean ± SD	3.42 ± 1.40	4.76 ± 14.64	4.39 ± 2.21	5.23 ± 3.22	4.45 ± 7.63	
Median (IQR)	3.20 (2.46, 4.12)	3.63 (2.89, 4.65)	3.90 (3.03, 5.23)	4.27 (2.99, 6.56)	3.68 (2.82, 4.99)	
Platelets						<0.001
Mean ± SD	246.01 ± 100.24	255.82 ± 80.97	266.92 ± 95.41	270.39 ± 104.87	259.78 ± 96.15	
Median (IQR)	232.50 (190.00, 278.50)	242.00 (200.50, 296.00)	255.50 (204.25, 310.50)	254.00 (201.00, 324.75)	244.50 (198.00, 303.00)	
Fibrinogen						<0.001
Mean ± SD	2.84 ± 0.72	3.30 ± 0.87	3.55 ± 0.94	3.90 ± 1.20	3.40 ± 1.02	
Median (IQR)	2.75 (2.33, 3.24)	3.24 (2.70, 3.80)	3.48 (2.90, 4.13)	3.66 (3.06, 4.71)	3.24 (2.68, 3.92)	
White Blood Cells						<0.001
Mean ± SD	5.58 ± 1.67	7.19 ± 16.47	6.69 ± 2.55	7.44 ± 3.41	6.73 ± 8.57	
Median (IQR)	5.38 (4.43, 6.51)	6.01 (4.90, 7.12)	6.30 (5.16, 7.83)	6.42 (5.09, 8.84)	6.00 (4.88, 7.52)	
CEA						0.005
Mean ± SD	22.60 ± 91.15	63.18 ± 315.77	128.25 ± 965.11	42.93 ± 180.74	64.24 ± 518.56	
Median (IQR)	3.83 (2.13, 7.88)	4.51 (2.57, 11.97)	4.57 (2.25, 17.48)	4.80 (2.48, 11.88)	4.51 (2.31, 11.84)	
HDL-C						<0.001
Mean ± SD	1.18 ± 0.32	1.12 ± 0.30	1.02 ± 0.28	0.95 ± 0.31	1.07 ± 0.31	
Median (IQR)	1.13 (0.95, 1.37)	1.07 (0.90, 1.25)	0.99 (0.83, 1.18)	0.92 (0.74, 1.14)	1.03 (0.86, 1.23)	
LDL-C						0.001
Mean ± SD	2.54 ± 0.70	2.77 ± 0.78	2.75 ± 0.94	2.64 ± 0.99	2.68 ± 0.86	
Median (IQR)	2.50 (2.06, 2.94)	2.76 (2.27, 3.18)	2.65 (2.19, 3.27)	2.59 (2.05, 3.15)	2.63 (2.14, 3.13)	
**eGFR**						<0.001
Mean ± SD	89.80 ± 16.60	84.98 ± 18.50	84.73 ± 19.24	80.41 ± 25.32	84.98 ± 20.43	
Median (IQR)	93.46 (81.32, 100.33)	88.57 (74.16, 97.07)	88.48 (75.35, 96.84)	86.24 (67.38, 96.81)	89.64 (74.71, 98.27)	
**SBP**						0.026
Mean ± SD	120.76 ± 17.09	124.45 ± 18.26	124.43 ± 19.83	125.47 ± 60.50	123.78 ± 34.21	
Median (IQR)	119.50 (110.00, 131.00)	124.50 (112.00, 136.00)	125.00 (111.00, 137.00)	122.00 (111.00, 132.75)	123.00 (111.00, 134.00)	
**DBP**						0.034
Mean ± SD	76.14 ± 11.10	78.47 ± 11.01	78.62 ± 12.62	77.34 ± 12.16	77.64 ± 11.77	
Median (IQR)	76.00 (69.00, 82.75)	79.00 (71.00, 86.00)	78.00 (69.25, 87.00)	77.00 (70.00, 84.00)	77.00 (70.00, 85.00)	
**BMI**						0.179
Mean ± SD	21.90 ± 3.31	22.46 ± 3.14	22.37 ± 3.54	22.07 ± 3.42	22.20 ± 3.36	
Median (IQR)	21.64 (19.53, 24.02)	22.41 (20.33, 24.22)	21.97 (19.99, 24.15)	22.05 (19.82, 24.22)	22.02 (19.91, 24.17)	
**Hypertension**						0.013
No	256 (88.3%)	244 (84.1%)	227 (78.3%)	243 (83.8%)	970 (83.6%)	
Yes	34 (11.7%)	46 (15.9%)	63 (21.7%)	47 (16.2%)	190 (16.4%)	
**Diabetes**						0.002
No	286 (98.6%)	272 (93.8%)	267 (92.1%)	268 (92.4%)	1,093 (94.2%)	
Yes	4 (1.4%)	18 (6.2%)	23 (7.9%)	22 (7.6%)	67 (5.8%)	
Malnutrition						0.032
No	260 (89.7%)	270 (93.1%)	248 (85.5%)	258 (89.0%)	1,036 (89.3%)	
Yes	30 (10.3%)	20 (6.9%)	42 (14.5%)	32 (11.0%)	124 (10.7%)	
**Surgery**						<0.001
No	119 (41.0%)	142 (49.0%)	164 (56.6%)	172 (59.3%)	597 (51.5%)	
Yes	171 (59.0%)	148 (51.0%)	126 (43.4%)	118 (40.7%)	563 (48.5%)	
**Chemotherapy**						<0.001
No	90 (31.0%)	123 (42.4%)	126 (43.4%)	136 (46.9%)	475 (40.9%)	
Yes	200 (69.0%)	167 (57.6%)	164 (56.6%)	154 (53.1%)	685 (59.1%)	

CTI, C-reactive protein-triglyceride-glucose index; CRP, C-reactive protein; TyG, triglyceride-glucose index; HDL-C, high-density lipoprotein cholesterol; LDL-C, low-density lipoprotein cholesterol; eGFR, estimated glomerular filtration rate; SBP, systolic blood pressure; DBP, diastolic blood pressure; BMI, body mass index; CEA, carcinoembryonic antigen; IQR, interquartile range; SD, standard deviation; ANOVA, analysis of variance.

Several inflammatory and metabolic parameters showed significant differences across CTI quartiles. Compared with the Q1 group, patients in the Q4 group had significantly higher levels of CRP, TyG, white blood cell count, neutrophil count, and fibrinogen (all *p* < 0.001), while serum albumin and high-density lipoprotein cholesterol (HDL-C) levels were significantly lower (all *p* < 0.001). Regarding clinical characteristics, patients in the higher CTI groups had higher prevalence of hypertension (16.2% vs. 11.7%, *p* = 0.013) and diabetes mellitus (7.6% vs. 1.4%, *p* = 0.002), and were older on average (*p* = 0.016). The proportions of patients receiving surgery and chemotherapy were lower in the higher CTI quartile groups compared with the lower quartile groups (both *p* < 0.001).

The independent UK Biobank validation cohort included 1,752 patients with rectal cancer, among whom 442 developed incident cardiovascular events during follow-up. The distribution of baseline characteristics in this population was generally consistent with that of the primary analysis cohort, as detailed in Supplementary Table S2.

### 3.2 Association between CTI and risk of incident CVD

Multivariable logistic regression analysis demonstrated a statistically significant association between CTI and the risk of incident CVD (Table 2). After full adjustment for potential confounders including age, sex, BMI, history of hypertension, history of diabetes, eGFR, history of surgery, and history of chemotherapy (Model 3), each one-standard-deviation (SD) increase in CTI was associated with an 18% increased risk of incident CVD (OR = 1.18, 95% CI: 1.02–1.36, *p* = 0.025). When CTI was analyzed as a categorical variable, a significantly increased risk of incident CVD was observed only among patients in the highest quartile (Q4) compared with those in the lowest quartile (Q1) (OR = 1.60, 95% CI: 1.07–2.42, *p* = 0.024), whereas no statistically significant associations were observed for the intermediate quartiles (Q2 and Q3), despite an overall statistical trend (*p* for trend = 0.034).

**Table 2:** Association between CTI and risk of incident CVD in rectal cancer survivors.

**Variable**	**Model 1**		**Model 2**		**Model 3**	
	**OR (95% CI)**	***P***	**OR (95% CI)**	***P***	**OR (95% CI)**	***P***
Per SD increase	1.20 (1.05, 1.38)	0.009	1.15 (1.00, 1.33)	0.054	1.18 (1.02, 1.36)	0.025
**Q1**	**1.00** **(Ref)**	**-**	**1.00** **(Ref)**	**-**	**1.00** **(Ref)**	**-**
**Q2**	1.48 (1.00, 2.21)	0.053	1.39 (0.93, 2.09)	0.113	1.44 (0.96, 2.18)	0.081
**Q3**	1.57 (1.06, 2.34)	0.026	1.38 (0.92, 2.08)	0.118	1.47 (0.98, 2.23)	0.065
**Q4**	1.64 (1.11, 2.44)	0.014	1.50 (1.00, 2.26)	0.050	1.60 (1.07, 2.42)	0.024
***P* for Trend**	0.018		0.072		0.034	

Model 1: adjusted for age, sex, and BMI. Model 2: additionally adjusted for history of hypertension, history of diabetes, and eGFR. Model 3 (fully adjusted): further adjusted for history of surgery and history of chemotherapy. CTI, C-reactive protein-triglyceride-glucose index; CVD, cardiovascular disease; OR, odds ratio; CI, confidence interval; SD, standard deviation; Ref, reference; BMI, body mass index; eGFR, estimated glomerular filtration rate.

Bold values indicate the reference category.

To further characterize the mechanism-specific associations, secondary exploratory analyses were conducted across the major cardiovascular subgroups (Supplementary Table S3). In the fully adjusted categorical analysis, a statistically significant association between CTI and incident CVD was observed for cardiac dysfunction-related events (*n* = 112; OR = 1.369, 95% CI: 1.11–1.69, *p* = 0.003). No statistically significant associations were observed for thromboembolic and pulmonary vascular events (OR = 1.22, 95% CI: 0.93–1.60, *p* = 0.147) or atherosclerotic vascular events (OR = 0.91, 95% CI: 0.74–1.12, *p* = 0.370).

### 3.3 Dose–response relationship between CTI and risk of incident CVD

**Figure 2:**
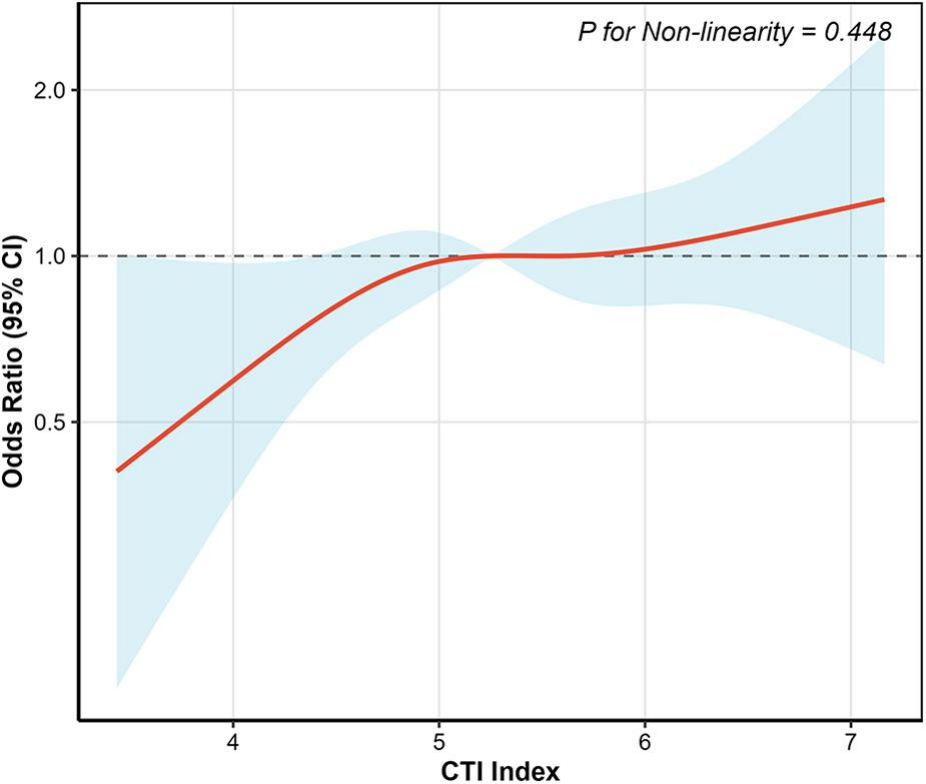
Dose–response relationship between CTI and risk of incident cardiovascular disease. The solid blue line represents the odds ratio (OR) for CVD, with CTI was modeled as a continuous variable using restricted cubic splines (RCS) with four knots. The shaded area indicates the 95% confidence interval. The dashed horizontal line at OR = 1.0 serves as the reference. CTI, C-reactive protein-triglyceride-glucose index; CVD, cardiovascular disease; OR, odds ratio; CI, confidence interval; RCS, restricted cubic spline.

In the fully adjusted model, RCS were employed to further characterize the dose–response relationship between CTI and the risk of incident CVD (Figure 2). The analysis demonstrated an approximately linear positive association between continuous CTI and incident CVD risk (*p* for non-linearity = 0.448). Wider confidence intervals were observed at the extremes of the CTI distribution. Stratified RCS analyses showed that the positive association was observed across different subgroups (Supplementary Figure S2).

### 3.4 Predictor selection and evaluation of incremental value of CTI

LASSO regression was used for variable selection and dimensionality reduction. Under the optimal penalty parameter *λ*, CTI, along with 11 other variables including history of hypertension, history of diabetes, age, BMI, and history of surgery, were retained together, constituting the final set of candidate predictors selected for model construction (Supplementary Figure S3).

**Figure 3:**
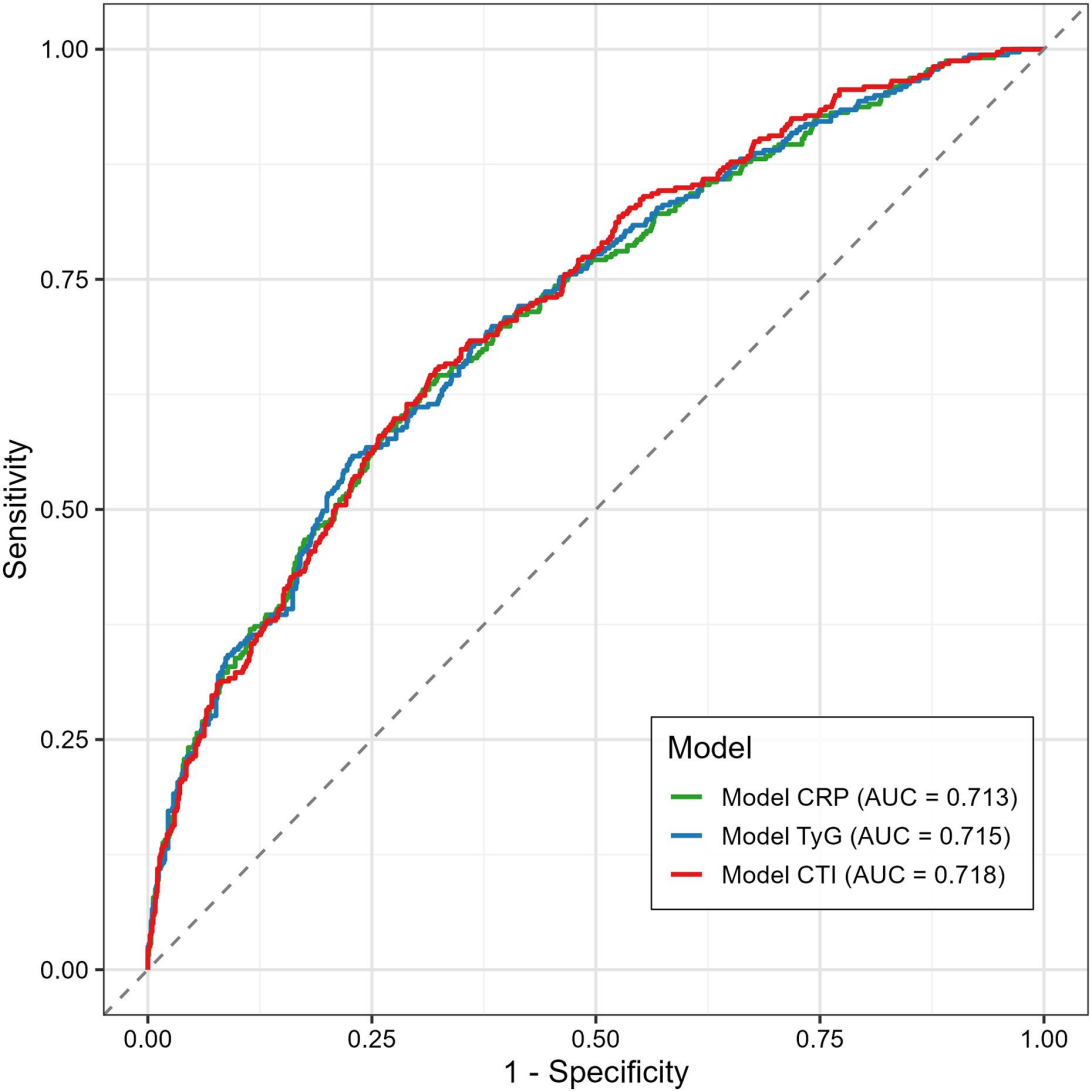
Receiver operating characteristic curves for CTI, TyG, and CRP in predicting incident cardiovascular disease. Receiver operating characteristic (ROC) curves comparing the discriminative ability of three multivariable models for predicting incident cardiovascular disease (CVD) in rectal cancer survivors (*n* = 1,160). All models were adjusted for the same covariates as in Model 3 of Table 2 (age, sex, body mass index, history of hypertension, history of diabetes, estimated glomerular filtration rate, history of surgery, and history of chemotherapy), with the addition of different inflammatory–metabolic markers: C-reactive protein (CRP) alone (Model 1, green line), triglyceride–glucose index (TyG) alone (Model 2, blue line), and the C-reactive protein–triglyceride–glucose index (CTI) (Model 3, red line). The diagonal dashed line represents the line of no discrimination (AUC = 0.5). CTI, C-reactive protein–triglyceride–glucose index; TyG, triglyceride–glucose index; CRP, C-reactive protein; CVD, cardiovascular disease; ROC, receiver operating characteristic; AUC, area under the receiver operating characteristic curve; CI, confidence interval.

We further evaluated the incremental value of CTI in multivariable models (Table 3). Upon incorporating CTI into the fully adjusted multivariable model, the model demonstrated improved goodness-of-fit, with the −2 log-likelihood (−2LL) and AIC decreasing to their lowest values (1,212.75 and 1,236.75, respectively). The AUC increased to 0.718 (95% CI: 0.69–0.75). Specifically, DeLong's test indicated that the AUC of the CTI-incorporated model (0.718) was significantly higher than that of the model incorporating CRP alone (Model 1: AUC = 0.713, *p* = 0.050) or TyG alone (Model 2: AUC = 0.715, *p* = 0.042) (Figure 3). Calibration performance and decision curve analysis results for the models are presented in Supplementary Figures S4. In further analyses evaluating risk reclassification, the addition of CTI to the baseline multivariable clinical model demonstrated statistically significant improvements in risk reclassification (Table 4), with a categorical NRI of 0.052 (95% CI: 0.018–0.086, *p* = 0.005) and an IDI of 0.004 (95% CI: 0.001–0.007, *p* = 0.015). When evaluated against individual components, the CTI-inclusive model demonstrated statistically significant improvements in reclassification and discrimination compared with the CRP-alone model (NRI = 0.038, 95% CI: 0.008–0.068, *p* = 0.018; IDI = 0.002, 95% CI: 0.000–0.005, *p* = 0.048); improvements compared with the TyG-alone model did not reach conventional statistical significance (NRI: *p* = 0.053; IDI: *p* = 0.072).

**Table 3:** Incremental discriminative performance of CTI compared with individual biomarkers for incident CVD.

Model	Added Variable	−2LL	AIC	AUC (95% CI)	*P* value^a^
Model 1	CRP	1,217.00	1,241.00	0.713 (0.679-0.746)	0.050
Model 2	TyG	1,214.65	1,238.65	0.715 (0.682-0.748)	0.042
Model 3	CTI	1,212.75	1,236.75	0.718 (0.686-0.751)	*Reference*

CRP, C-reactive protein; TyG, triglyceride-glucose index; CTI, C-reactive protein-triglyceride-glucose index; −2LL, −2 Log Likelihood; AIC, Akaike Information Criterion; AUC, Area under the receiver operating characteristic curve; CI, Confidence interval.

a P-values represent comparisons of each model's AUC against that of Model 3 (CTI) using DeLong's test; Model 3 serves as the reference.

**Table 4:** Incremental predictive value of CTI beyond baseline clinical models and individual components.

Comparison Models	**Categorical NRI (95% CI)**	***P***	**IDI (95% CI)**	***P***
Base + CTI vs. Base model	0.052 (0.018–0.086)	0.005	0.004 (0.001–0.007)	0.015
Base + CTI vs. Base + CRP	0.038 (0.008–0.068)	0.018	0.002 (0.000–0.005)	0.048
Base + CTI vs. Base + TyG	0.028 (0.001–0.055)	0.053	0.001 (0.000–0.003)	0.072

The base model included age, sex, body mass index, history of hypertension, history of diabetes, eGFR, history of surgery, and history of chemotherapy. NRI: Net Reclassification Improvement; IDI: Integrated Discrimination Improvement; CI: Confidence Interval.

### 3.5 Multivariable model performance and internal and external validation

To develop a risk prediction model for incident CVD, we compared the predictive performance of various machine learning algorithms, including GLM, KNN, RF, and SVM (Supplementary Table S4). GLM demonstrated stable performance across validation metrics and was selected as the final model.

In the primary analysis cohort, the apparent AUC of the GLM model was 0.718, which decreased to 0.704 after internal validation using bootstrap resampling. Calibration curves showed good agreement between predicted risks and observed probabilities (Hosmer-Lemeshow test *p* = 0.80), with a model optimism of 0.014 (Table 5). In the independent UK Biobank validation cohort, the model achieved an AUC of 0.649. After recalibration of the intercept and slope, the calibration performance improved, with the Brier score decreasing from 0.181 to 0.179.

**Table 5:** Performance of the predictive model in the development and external validation cohorts.

**Cohort**	**Metric**	**Value**
Development cohort	AUC (95% CI)	0.718 (0.686–0.751)
	Hosmer–Lemeshow test	*χ*^2^ = 4.595, *p* = 0.800
	Brier score	0.181
	Bootstrap-corrected AUC	0.704
External validation cohort	AUC (95% CI)	0.649 (0.620–0.679)
	Hosmer–Lemeshow test^a^	χ^2^ = 5.059, *p* = 0.280
	Brier score	0.179

a The Hosmer-Lemeshow test in the external validation cohort was performed after recalibration of the model intercept and slope to account for differences in baseline risk between cohorts.

AUC, area under the receiver operating characteristic curve; CI, confidence interval; χ², chi-square.

### 3.6 Model interpretability analysis

**Figure 4:**
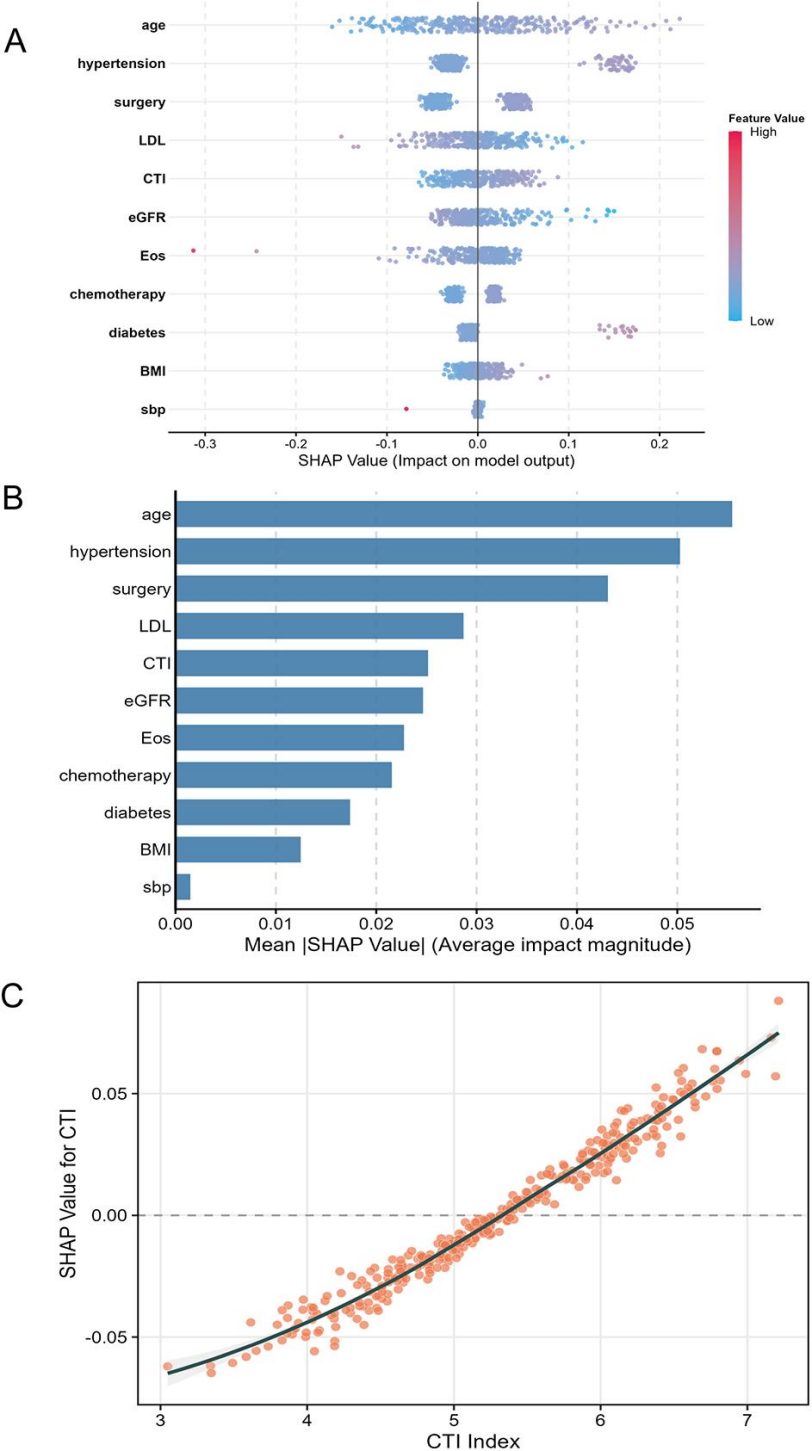
SHAP-Based interpretation of predictor contributions in the final prediction model. **(A)** SHAP summary plot displaying the distribution of feature contributions to the predictive model for incident cardiovascular disease. Each point represents the SHAP value for a given feature and an individual patient; the color indicates the actual feature value (red: high, blue: low). Positive SHAP values indicate a higher predicted risk. **(B)** SHAP feature importance bar plot displaying the mean absolute SHAP values for each variable, representing the average magnitude of impact on the model output. **(C)** SHAP dependence plot illustrating the relationship between CTI values and their corresponding SHAP contributions within the fitted model. SHAP, SHapley Additive exPlanations; CTI, C-reactive protein-triglyceride-glucose index; LDL-C, low-density lipoprotein cholesterol; eGFR, estimated glomerular filtration rate; Eos, eosinophil count; BMI, body mass index; SBP, systolic blood pressure.

**Figure 5:**
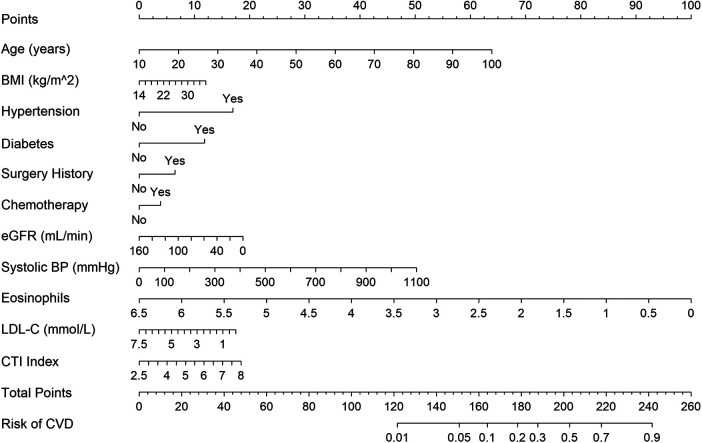
Nomogram for predicting incident cardiovascular disease in rectal cancer survivors. Nomogram constructed based on the final multivariable logistic regression model incorporating LASSO-selected and clinically relevant predictors for predicting the probability of incident cardiovascular disease (CVD) in rectal cancer survivors. The model includes the following predictors: age, history of hypertension, history of surgery, low-density lipoprotein cholesterol (LDL-C), C-reactive protein-triglyceride-glucose index (CTI), estimated glomerular filtration rate (eGFR), eosinophil count, history of chemotherapy, history of diabetes, body mass index (BMI), and systolic blood pressure (SBP). CVD, cardiovascular disease; CTI, C-reactive protein-triglyceride-glucose index; LDL-C, low-density lipoprotein cholesterol; eGFR, estimated glomerular filtration rate; BMI, body mass index; SBP, systolic blood pressure.

SHAP analysis showed that age, history of hypertension, history of surgery, LDL-C, and CTI had the highest relative contributions within the final model (Figures 4A,B). The SHAP dependence plot indicated a positive relationship between CTI values and their corresponding SHAP contributions within the fitted model (Figure 4C). A nomogram based on the final model was constructed to facilitate individualized risk estimation (Figure 5).

### 3.7 Subgroup analyses, sensitivity analyses, and attenuation effect analysis

**Figure 6:**
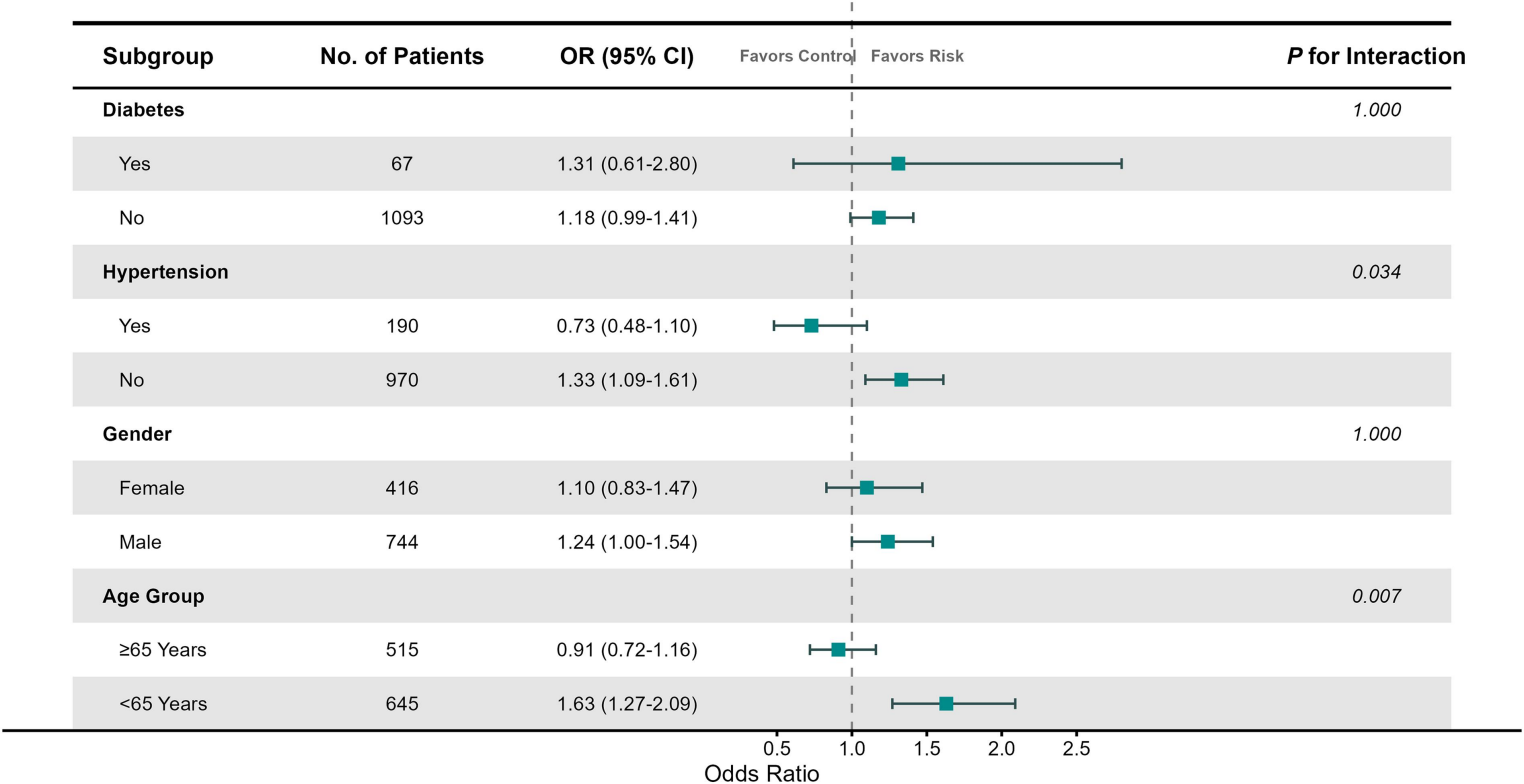
Subgroup analysis for the association between CTI and incident cardiovascular disease. Forest plot displaying the association between the C-reactive protein–triglyceride–glucose index (CTI) and the risk of incident cardiovascular disease (CVD) across prespecified subgroups in rectal cancer survivors (*n* = 1,160). Odds ratios (OR) and 95% confidence intervals (CI) were derived from multivariable logistic regression models adjusted for the same covariates as in Model 3 of Table 2, except for the stratification variable itself. CTI was modeled as a continuous variable (per standard deviation increase). Each square represents the point estimate of the OR for the specified subgroup, with horizontal lines indicating the 95% CI. The size of the square is proportional to the number of patients in that subgroup. The vertical dashed line at OR = 1.0 represents the null value (no association). The *P* value for interaction for each subgroup stratum is displayed in the rightmost column of the forest plot, indicating whether the association between CTI and CVD differs significantly across subgroup categories. CTI, C-reactive protein-triglyceride-glucose index; CVD, cardiovascular disease; OR, odds ratio; CI, confidence interval.

Subgroup analyses revealed significant effect modification in the association between CTI and incident CVD risk across different clinical strata (Figure 6). The risk association was more pronounced in patients aged < 65 years (OR = 1.63, 95% CI: 1.27–2.09) compared to those aged ≥65 years (OR = 0.91, 95% CI: 0.72–1.16; *p* for interaction = 0.007). The association was also more pronounced in patients without a history of hypertension (OR = 1.33, 95% CI: 1.09–1.61) than in those with established hypertension (OR = 0.73, 95% CI: 0.48–1.10; *p* for interaction = 0.034).

Results from multiple sensitivity analyses were consistent with the primary findings (Table 6). After excluding underweight individuals (BMI < 18.5 kg/m^2^), the positive association between CTI and CVD risk remained significant (OR = 1.26, 95% CI: 1.04–1.52, *p* = 0.017). When excluding patients with baseline hypertension or diabetes, the association remained statistically significant with a larger effect estimate (OR = 1.31, 95% CI: 1.08–1.59, *p* = 0.007), with a calculated E-value of 1.95. After excluding the 461 participants with baseline CRP > 10 mg/L, the point estimate remained directionally consistent (OR per SD increase = 1.292, 95% CI: 0.951–1.768, *p* = 0.105), though statistical significance was not reached, likely reflecting reduced statistical power in this restricted sample (Supplementary Table S5).

**Table 6:** Sensitivity analyses for the association between CTI and incident CVD risk.

**Analysis**	**N**	**Events**	**OR (95% CI)**	***P***	**E-value**
Exclude Underweight (BMI < 18.5)	1,001	283	1.26 (1.04–1.52)	0.017	1.82
Exclude Elderly (Age > 75)	1,005	240	1.19 (0.99–1.43)	0.068	1.66
Exclude Baseline HTN/DM	932	204	1.31 (1.08–1.59)	0.007	1.95

All analyses were performed using the fully adjusted multivariable logistic regression model (Model 3), adjusted for age, sex, BMI, history of hypertension, history of diabetes, eGFR, history of surgery, and history of chemotherapy. CTI, C-reactive protein-triglyceride-glucose index; CVD, cardiovascular disease; BMI, body mass index; HTN, hypertension; DM, diabetes mellitus; OR, odds ratio; CI, confidence interval.

Finally, an attenuation of the CTI–CVD association was observed in the UK Biobank validation cohort (Table 7). While CTI showed a significant association in the unadjusted model (OR = 1.23, 95% CI:1.10–1.37, *p* < 0.001), the effect estimate gradually diminished and lost statistical significance after sequential adjustment for demographics (age, sex), lifestyle factors (BMI, smoking, alcohol), comorbidities (hypertension, diabetes), renal function (eGFR), and treatment history (surgery, chemotherapy) (Model 5: OR = 1.08, 95% CI:0.95–1.22, *p* = 0.261).

**Table 7:** Attenuation of the CTI–CVD association after sequential adjustment in the UK biobank validation cohort.

**Model**	**Adjustments added**	***β* (log OR)**	**OR (95% CI)**	***P***
Model 0	CTI only	0.204	1.23 (1.10–1.37)	<0.001
Model 1	+ Age, Sex	0.16	1.17 (1.05–1.31)	0.005
Model 2	+ BMI, Smoking, Drinking	0.082	1.09 (0.96–1.23)	0.199
Model 3	+ Hypertension, Diabetes	0.077	1.08 (0.95–1.22)	0.234
Model 4	+ eGFR	0.071	1.07 (0.95–1.22)	0.271
Model 5	+ Surgery, Chemotherapy	0.073	1.08 (0.95–1.22)	0.261

CTI, C-reactive protein-triglyceride-glucose index; CVD, cardiovascular disease; OR, odds ratio; CI, confidence interval; BMI, body mass index; eGFR, estimated glomerular filtration rate.

## 4 Discussion

This is among the first studies to examine the association between CTI—a composite index integrating inflammatory and metabolic signals—and incident CVD in rectal cancer survivors. This question addresses a gap in the cardio-oncology literature: how to capture residual cardiovascular risk arising from cancer- and treatment-related processes that extend beyond traditional risk factors and pre-existing chronic conditions. We found that CTI was independently associated with incident CVD, with each one-SD increment conferring an 18% higher risk after full multivariable adjustment (OR = 1.18, 95% CI: 1.02–1.36). Because this association persisted after accounting for age, hypertension, diabetes, and other metabolic covariates, CTI appears to encode prognostic information that conventional risk factors do not fully capture. In this study, CTI was evaluated as a predictor of cumulative incident cardiovascular disease within the defined observation window rather than as a predictor of time-to-event outcomes; the reported associations therefore reflect cumulative risk over follow-up rather than hazard-based estimates.

The magnitude of this association was nonetheless modest. Categorical analyses showed that the excess risk was concentrated in the highest CTI quartile, with limited risk separation across the intermediate categories, suggesting that CTI is most informative for flagging patients with pronounced inflammatory–metabolic derangement rather than for graded discrimination across the full risk spectrum. The association was also stronger in younger patients and in those without baseline hypertension, and it attenuated in fully adjusted analyses within the UK Biobank cohort—findings we examine in the following sections.

### 4.1 Biological rationale for a composite inflammatory-metabolic indicator

Systemic inflammation and insulin resistance are well established as key pathological mediators involved in CVD development and progression. Recent large-scale prospective cohort studies have shown that elevated high-sensitivity C-reactive protein (hsCRP) levels are significantly associated with major adverse cardiovascular events and cardiovascular mortality, with residual inflammatory risk persisting even among populations achieving optimal low-density lipoprotein cholesterol control (23). In parallel, recent investigations have confirmed that the TyG index serves not only as a stable surrogate marker of insulin resistance but is also significantly associated with coronary heart disease, chronic kidney disease, and major adverse cardiovascular events (MACE) in multimorbid populations (13, 16). The cardiovascular relevance of combining inflammation-related markers has further been validated in large general-population cohorts (24). Taken together, these findings support the notion that inflammatory activation and metabolic dysregulation constitute a shared pathological mechanism underlying CVD, and their combined assessment may enable more refined cardiovascular risk stratification.

However, the aforementioned studies have predominantly focused on non-cancer populations or individuals with stable coronary artery disease, leaving an important evidence gap regarding cancer survivors—a distinct population characterized by prolonged exposure to heightened inflammatory and metabolic stress. Our findings extend recent epidemiological observations highlighting the importance of inflammatory-metabolic interactions in cardiovascular risk. Prior work has demonstrated that the interplay between lipid metabolism and inflammation is associated with increased cardiovascular risk in populations with metabolic abnormalities (25). The magnitude of association observed in our study (OR = 1.18 per SD increase in CTI) is broadly consistent with effect estimates reported in these prior studies, although direct numerical comparison is limited by differences in study populations, exposure definitions, and outcome ascertainment methods. Extending this concept into the cardio-oncology setting, our study suggests that the integrated inflammatory-metabolic status captured by CTI may provide additional information regarding CVD risk among rectal cancer survivors. Higher CTI levels were also accompanied by a clustering of adverse features, including elevated procoagulant markers, declining nutritional status, and dyslipidemia, in line with prior evidence linking cancer-related chronic inflammation and metabolic disturbances to accelerated atherosclerosis (25, 26). Compared with traditional strategies relying on single inflammatory or metabolic markers, CTI integrates CRP and TyG information into a composite index, potentially providing a broader representation of multifactorial risk profiles (14, 27). This composite biomarker approach is consistent with emerging research directions in CVD risk assessment that combine multiple biomarkers, metabolomic profiles, and genetic risk scores (9). From a cardio-oncology perspective, the inflammation-metabolism axis reflected by CTI is consistent with mechanisms proposed in the “cancer-heart axis” framework, which emphasizes systemic inflammation and metabolic stress as shared drivers of cardiovascular pathology (28–31).

A methodological consideration raised by the reliance on CRP is whether CTI primarily captures cardiovascular risk or acts as a passive surrogate for underlying cancer severity and treatment intensity. Because CRP is an acute-phase reactant heavily influenced by tumor-related inflammation, baseline CTI values expectedly incorporate signals of oncological stress. However, systemic inflammatory activation and metabolic dysregulation are increasingly recognized as active contributors to cardiovascular injury rather than merely reflecting tumor progression in isolation. Previous studies, including work from our group, have demonstrated that in rectal cancer survivors, both elevated systemic inflammation (18) and insulin resistance abnormalities (32) are associated with subsequent adverse cardiovascular complications. Therefore, by combining these synergistic mechanisms, CTI may be conceptualized as a pragmatic marker designed to reflect the joint cardiometabolic stress and vulnerability unique to the oncology setting, rather than a conventional indicator of cancer burden.

Our secondary exploratory analyses suggested that the association between CTI and CVD risk was predominantly observed for cardiac dysfunction-related events (such as heart failure and arrhythmias; OR = 1.37, *p* = 0.003), rather than classical atherosclerotic pathways (OR = 0.91, *p* = 0.370) or thromboembolic events (OR = 1.22, *p* = 0.147). This observation may reflect differences in cardiovascular risk patterns between cancer survivors and general populations. In rectal cancer survivors, treatment-related cardiotoxicity—including fluorouracil-induced vasospasm, surgical stress-induced neurohormonal activation, and radiation-mediated myocardial fibrosis—may preferentially induce direct myocardial injury and electrical instability rather than accelerating chronic lipid-driven atherogenesis. The systemic inflammatory activation and insulin resistance captured by CTI are mechanistically more compatible with these acute-to-subacute myocardial stress pathways, potentially explaining why the CTI–CVD association in this cohort was predominantly observed for cardiac dysfunction-related events rather than atherosclerotic endpoints. The non-significant inverse association observed for atherosclerotic events warrants cautious interpretation and likely reflects the relatively short follow-up window, which is insufficient to capture the slow progression of classical atherosclerosis in this population.

### 4.2 Subgroup differences and early risk identification

The association between CTI and incident CVD risk was more pronounced in patients aged under 65 years and in those without a baseline history of hypertension (both *p* for interaction <0.05). Large-scale population studies and risk assessment models suggest that in older individuals or those with established hypertension, age and long-term blood pressure burden often dominate the risk architecture, limiting the relative contribution of novel inflammatory or metabolic markers (33, 34). Our findings align with this understanding: in older patients or those with hypertension, cumulative long-term vascular structural changes may overshadow the relative effects of inflammation–metabolism pathways, which may partly explain the weaker relative association observed in these subgroups.

Conversely, in rectal cancer survivors with a lower burden of traditional risk factors and no established history of hypertension or diabetes, an elevated CTI is more likely to represent an integrated reflection of subclinical vascular injury, persistent cancer-related inflammatory stress, and treatment-induced metabolic disturbances. This observation supports recent studies highlighting the role of non-traditional risk factors in shaping CVD risk among cancer survivors (35, 36). Thus, the clinical utility of CTI in this population may be best positioned as an early risk identification tool: complementing traditional risk frameworks centered on age and medical history by identifying a subset of otherwise unidentified high-risk individuals, thereby informing more nuanced follow-up and management strategies.

### 4.3 External validation and population context dependence

In the UK Biobank cohort, the positive association between CTI and CVD risk was significant in unadjusted models but gradually attenuated to non-significance after sequential adjustment for demographic characteristics, traditional cardiovascular risk factors, and prior cardiovascular history. This phenomenon reflects the well-documented healthy volunteer bias in the UK Biobank, characterized by a generally lower burden of traditional cardiovascular risk factors (37, 38). In such community-dwelling populations, the absolute risk architecture of incident CVD is predominantly established by classical determinants, including advanced age, chronic blood pressure burden, and long-standing metabolic comorbidities.

As demonstrated, the attenuation analysis revealed that a substantial proportion of the risk variance captured by CTI overlaps with established cardiovascular risk determinants in general populations, where age, hypertension, and metabolic comorbidities already account for a large proportion of the baseline risk architecture. These findings suggest that CTI should be considered a complementary inflammatory-metabolic marker rather than a replacement for established risk assessment approaches. Conversely, its independent contribution may become more apparent in selected cardio-oncology populations, where cancer survivors face overlapping influences from tumor-related inflammation, treatment-related stress, and metabolic disturbances.

In specialized oncology cohorts, however, evidence from real-world cancer survivor data has demonstrated that cancer survivors, particularly those who have received systemic anticancer therapy, have a significantly elevated risk of CVD compared with the non-cancer population, with marked heterogeneity observed across tumor types and treatment modalities (39–41). Recent reviews and consensus documents have further emphasized that chronic inflammation and metabolic disturbances constitute key pathological pathways linking cancer therapy to cardiovascular toxicity (42, 43). In such populations characterized by high inflammatory and metabolic burden, the inflammatory and metabolic signals integrated within CTI are more likely to be exacerbated, translating into observable differences in cardiovascular risk. These findings highlight the population-dependent nature of CTI-based risk assessment and underscore the need for population-specific validation when applying CTI across different clinical settings, given that differences in baseline risk profiles and cancer-related characteristics may influence model transportability.

### 4.4 Clinical implications and applications in risk stratification

In clinical practice, accumulating evidence has established that CVD has emerged as a leading cause of non-cancer mortality among cancer survivors during long-term follow-up (44, 45). However, current follow-up strategies remain predominantly reliant on traditional risk factors such as age, history of hypertension, and diabetes, with insufficient attention paid to inflammation-metabolism-related residual risk. CTI, derived from routinely available laboratory parameters, modestly improved model fit and significantly enhanced risk reclassification in the primary cohort, offering a potentially accessible tool for cardiovascular risk stratification in rectal cancer survivors. This finding echoes emerging research directions that advocate for the integration of multiple biomarkers and metabolomic scores alongside traditional risk factors to enhance CVD risk stratification (46–51).

The integrated inflammatory-metabolic risk framework evaluated in this study is intended to assist in risk assessment, not to replace clinical judgment or directly guide specific treatment decisions. In practical application, an elevated CTI may help identify individuals who could benefit from closer cardiovascular monitoring, proactive management of metabolic abnormalities, or shortened follow-up intervals, particularly among rectal cancer survivors without established hypertension or diabetes who are nevertheless situated within a context of heightened inflammatory and metabolic burden (48, 49). For instance, rectal cancer survivors with CTI values in the highest quartile (>6.09 in our cohort) may represent a subgroup warranting structured cardiovascular surveillance, including periodic cardiac function assessment and proactive optimization of metabolic comorbidities, in accordance with the risk-stratified monitoring approach recommended in contemporary cardio-oncology guidelines (7, 8, 52, 53). Although further validation is required before clinical implementation, CTI may provide a convenient inflammatory-metabolic perspective for complementing cardiovascular risk assessment in selected high-risk oncology populations.

### 4.5 Strengths, limitations, and future research directions

The primary strength of this study lies in its systematic evaluation of CTI within a specific cardio-oncology cohort, providing population-level evidence for a composite inflammation-metabolism marker in a cardio-oncology setting where such integrated signals remain insufficiently characterized. The application of restricted cubic splines further demonstrated an approximately linear dose–response relationship without evidence of a threshold effect, reinforcing the biological plausibility of CTI as a continuous risk indicator. Moreover, comparative analysis with the large-scale UK Biobank cohort effectively delineated the context-dependent utility of this integrated signal.

Nevertheless, several limitations warrant consideration. The single-center retrospective design introduces inherent biases, and residual confounding from unmeasured oncological characteristics—such as detailed staging, treatment intensity, and disease recurrence—cannot be excluded; accordingly, CTI should be interpreted as a context-dependent inflammatory–metabolic marker associated with cardiovascular risk in this population, rather than as a biomarker whose prognostic value is fully independent of cancer severity and treatment burden. Furthermore, reliance on retrospective electronic medical records limits the temporal resolution of incident events, restricting the application of robust time-to-event survival analyses. It is also important to note that CRP is an acute-phase reactant; while sensitivity analyses excluding participants with elevated CRP levels (>10 mg/L) showed generally consistent trends, transient inflammatory influences at baseline cannot be entirely ruled out. Finally, our reliance on single baseline measurements precludes the assessment of longitudinal CTI trajectories over the follow-up period. Future prospective, multicenter cohorts incorporating serial monitoring, cardiac-specific biomarkers, and imaging parameters are essential to validate the utility of CTI across broader oncological settings and to integrate it into multidimensional risk assessment frameworks.

## 5 Conclusion

In rectal cancer survivors, a higher CTI was independently associated with cumulative incident CVD within a defined follow-up window and improved risk reclassification beyond CRP or TyG alone, with the association concentrated among younger patients and those without established hypertension. This signal weakened to non-significance in the community-based UK Biobank cohort once traditional risk factors were accounted for, indicating that the incremental value of CTI depends on the underlying inflammatory–metabolic burden of the population studied. CTI may therefore help refine cardiovascular surveillance within specialized cardio-oncology settings, but should not be adopted as a universal risk-stratification tool. Prospective, multicenter studies with serial measurements and cardiac-specific endpoints are needed to confirm its clinical utility.

## Data Availability

The original contributions presented in the study are included in the article/Supplementary Material, further inquiries can be directed to the corresponding author. The UK Biobank data used in this study are available from the UK Biobank upon reasonable request (https://www.ukbiobank.ac.uk/).
