# Supplementary material for: Association of the C-reactive protein–triglyceride–glucose index with the risk of incident cardiovascular disease in rectal cancer survivors
Source: Front Cardiovasc Med. 2026 Jul 29;13:1880389. doi: 10.3389/fcvm.2026.1880389 (PMC13461421; doi:10.3389/fcvm.2026.1880389)
Supplement: Supplementary file 1 [file Table1.docx]

Supplementary Document

**Supplementary Document 1.** Appendix 4 of the Approved Research Project from UK Biobank, confirming the study reference number (532253), title, and associated governance and ethics terms.
