# Supplementary material for: Association of the C-reactive protein–triglyceride–glucose index with the risk of incident cardiovascular disease in rectal cancer survivors
Source: Front Cardiovasc Med. 2026 Jul 29;13:1880389. doi: 10.3389/fcvm.2026.1880389 (PMC13461421; doi:10.3389/fcvm.2026.1880389)
Supplement: Supplementary file 2 [file Table2.docx]

Supplementary Material

# 1 Supplementary Figures and Tables

## 1.1 Supplementary Tables

**Supplementary Table 1. Missing data proportions for key exposure, outcome, and covariate variables in the analytical models**

| **Variable** | **Primary Cohort (%)** | **UK Biobank Cohort (%)** |
| --- | --- | --- |
| CRP | 0 | 0 |
| Triglycerides | 0 | 0 |
| Fasting glucose | 0 | 0 |
| Incident CVD (primary outcome) | 0 | 0 |
| History of hypertension | 0 | 0.1 |
| History of diabetes | 0 | 0.3 |
| Smoking history | — | 0.3 |
| Alcohol consumption | — | 0.1 |
| Albumin | 0 | 0 |
| Eosinophil count | 0.5 | 13.6 |
| Basophil count | 0.5 | 0 |
| Lymphocyte count | 0.5 | 3.1 |
| Neutrophil count | 0.5 | 0 |
| Platelet count | 0.5 | 0 |
| White blood cell count | 0.5 | 2.9 |
| eGFR | 1 | 0 |
| Fibrinogen | 3.2 | 0 |
| CEA | 4.9 | — |
| HDL-C | 9.6 | 0 |
| LDL-C | 9.6 | 0 |
| DBP | 9.7 | 0 |
| SBP | 11.2 | 0 |
| BMI | 21.6 | 0 |
| Malnutrition | 0 | — |

Note: — indicates variable not applicable, not collected, or not extracted in this specific cohort. Missing variables were imputed using MICE before multivariable analyses. MICE = Multiple Imputation by Chained Equations; CRP = C-reactive protein; CVD = cardiovascular disease; HDL-C = high-density lipoprotein cholesterol; LDL-C = low-density lipoprotein cholesterol; DBP = diastolic blood pressure; SBP = systolic blood pressure; BMI = body mass index; eGFR = estimated glomerular filtration rate; CEA = carcinoembryonic antigen.

**Supplementary Table 2. Baseline Characteristics of Rectal Cancer Survivors Stratified by CTI Quartiles in the UK Biobank Validation Cohort**

| **Variable** | **Q1** | **Q2** | **Q3** | **Q4** | **Total** | ***P*** |
| --- | --- | --- | --- | --- | --- | --- |
| **Age** | 60.00 (54.00-65.00) | 61.00 (56.00-65.75) | 61.00 (56.00-66.00) | 62.00 (56.00-66.00) | 61.00 (56.00-65.00) | 0.002 |
| **Gender** |  |  |  |  |  | <0.001 |
| Female | 189 (43.2%) | 168 (38.4%) | 149 (34.0%) | 130 (29.7%) | 636 (36.3%) |  |
| Male | 249 (56.8%) | 270 (61.6%) | 289 (66.0%) | 308 (70.3%) | 1116 (63.7%) |  |
| **Education level** |  |  |  |  |  | 0.004 |
| No | 134 (30.6%) | 145 (33.1%) | 176 (40.2%) | 174 (39.7%) | 629 (35.9%) |  |
| Yes | 304 (69.4%) | 293 (66.9%) | 262 (59.8%) | 264 (60.3%) | 1123 (64.1%) |  |
| **Hypertension** |  |  |  |  |  | <0.001 |
| No | 330 (75.3%) | 275 (62.8%) | 276 (63.0%) | 241 (55.0%) | 1122 (64.0%) |  |
| Yes | 108 (24.7%) | 163 (37.2%) | 162 (37.0%) | 197 (45.0%) | 630 (36.0%) |  |
| **Diabetes** |  |  |  |  |  | 0.001 |
| No | 420 (95.9%) | 410 (93.6%) | 401 (91.6%) | 390 (89.0%) | 1621 (92.5%) |  |
| Yes | 18 (4.1%) | 28 (6.4%) | 37 (8.4%) | 48 (11.0%) | 131 (7.5%) |  |
| **Smoking history** |  |  |  |  |  | <0.001 |
| No | 238 (54.3%) | 178 (40.6%) | 201 (45.9%) | 168 (38.4%) | 785 (44.8%) |  |
| Yes | 200 (45.7%) | 260 (59.4%) | 237 (54.1%) | 270 (61.6%) | 967 (55.2%) |  |
| **Alcohol consumption** |  |  |  |  |  | 0.121 |
| No | 32 (7.3%) | 23 (5.3%) | 29 (6.6%) | 41 (9.4%) | 125 (7.1%) |  |
| Yes | 406 (92.7%) | 415 (94.7%) | 409 (93.4%) | 397 (90.6%) | 1627 (92.9%) |  |
| **SBP** | 136.50 (121.50-152.38) | 139.50 (127.12-155.00) | 140.00 (128.00-153.50) | 143.50 (131.50-156.38) | 140.00 (127.00-154.00) | <0.001 |
| **DBP** | 81.50 (73.00-88.50) | 82.50 (75.50-89.50) | 82.50 (76.00-89.88) | 84.00 (76.50-91.50) | 82.50 (75.50-89.50) | <0.001 |
| **CTI** | 3.44 ± 0.25 | 3.94 ± 0.11 | 4.32 ± 0.12 | 4.88 ± 0.29 | 4.14 ± 0.57 | <0.001 |
| **BMI** | 24.43 (22.60-26.90) | 26.74 (24.66-29.08) | 28.10 (25.88-30.68) | 29.42 (26.79-32.89) | 27.20 (24.51-30.13) | <0.001 |
| **CRP** | 0.52 (0.34-0.78) | 1.13 (0.80-1.61) | 2.10 (1.47-3.05) | 4.92 (3.08-8.58) | 1.53 (0.77-3.10) | <0.001 |
| **Albumin** | 45.51 ± 2.55 | 45.16 ± 2.65 | 44.86 ± 2.60 | 44.59 ± 2.72 | 45.03 ± 2.65 | <0.001 |
| **Blood glucose** | 87.87 (81.71-93.67) | 90.12 (83.13-95.21) | 91.10 (84.40-99.30) | 93.39 (86.31-105.70) | 90.24 (83.72-97.99) | <0.001 |
| **Triglycerides** | 18.56 (14.68-24.43) | 26.31 (20.56-34.70) | 33.45 (25.41-43.82) | 43.23 (32.62-60.07) | 29.16 (20.38-40.98) | <0.001 |
| **HDL-C** | 1.56 (1.34-1.83) | 1.42 (1.20-1.74) | 1.33 (1.12-1.51) | 1.18 (1.01-1.39) | 1.36 (1.14-1.64) | <0.001 |
| **LDL-C** | 3.37 ± 0.81 | 3.50 ± 0.87 | 3.64 ± 1.00 | 3.60 ± 0.89 | 3.52 ± 0.90 | <0.001 |
| **eGFR** | 92.89 (83.97-99.14) | 91.26 (83.15-97.23) | 90.52 (79.02-96.78) | 91.25 (80.48-97.68) | 91.62 (81.66-97.57) | 0.0028 |
| **Eosinophil Count** | 0.10 (0.09-0.20) | 0.14 (0.10-0.23) | 0.17 (0.10-0.25) | 0.19 (0.10-0.28) | 0.15 (0.10-0.23) | <0.0001 |
| **Basophil Count** | 0.03 (0.02-0.05) | 0.03 (0.02-0.06) | 0.03 (0.02-0.05) | 0.03 (0.02-0.06) | 0.03 (0.02-0.06) | 0.1187 |
| **Lymphocyte Count** | 1.70 (1.40-2.06) | 1.77 (1.46-2.20) | 1.83 (1.53-2.27) | 1.90 (1.48-2.33) | 1.80 (1.45-2.20) | <0.0001 |
| **Neutrophil Count** | 3.66 (3.02-4.57) | 3.96 (3.21-4.80) | 4.10 (3.32-5.11) | 4.70 (3.74-5.61) | 4.04 (3.30-5.06) | <0.0001 |
| **White Blood Cells** | 6.17 (5.13-7.05) | 6.44 (5.59-7.60) | 6.70 (5.80-7.94) | 7.34 (6.16-8.69) | 6.60 (5.60-7.90) | <0.0001 |
| **Platelets** | 239.00 (204.72-269.23) | 242.90 (205.00-283.80) | 247.05 (214.18-282.08) | 250.00 (205.85-290.62) | 244.75 (207.67-282.00) | 0.0065 |
| **Surgery** |  |  |  |  |  | 0.969 |
| No | 428 (97.7%) | 426 (97.3%) | 426 (97.3%) | 427 (97.5%) | 1707 (97.4%) |  |
| Yes | 10 (2.3%) | 12 (2.7%) | 12 (2.7%) | 11 (2.5%) | 45 (2.6%) |  |
| **Chemotherapy** |  |  |  |  |  | 0.448 |
| No | 315 (71.9%) | 304 (69.4%) | 300 (68.5%) | 319 (72.8%) | 1238 (70.7%) |  |
| Yes | 123 (28.1%) | 134 (30.6%) | 138 (31.5%) | 119 (27.2%) | 514 (29.3%) |  |

Note: CTI, C-reactive protein-triglyceride-glucose index; SBP, systolic blood pressure; DBP, diastolic blood pressure; BMI, body mass index; CRP, C-reactive protein; HDL-C, high-density lipoprotein cholesterol; LDL-C, low-density lipoprotein cholesterol; eGFR, estimated glomerular filtration rate; IQR, interquartile range; SD, standard deviation.

**Supplementary Table 3**. **Association Between CTI and Different Categories of Incident Cardiovascular Events**

| **Outcome** | **Events** | **OR (95% CI)** | ***P*** |
| --- | --- | --- | --- |
| Overall cardiovascular events | 319 | 1.18 (1.02–1.36) | 0.025 |
| Cardiac dysfunction-related events | 112 | 1.37 (1.11–1.69) | 0.003 |
| Thromboembolic and pulmonary vascular events | 61 | 1.22 (0.93–1.60) | 0.147 |
| Atherosclerotic vascular events | 146 | 0.91 (0.74–1.12) | 0.370 |

Note: Values represent odds ratios (ORs) and 95% confidence intervals (CIs) for incident cardiovascular events associated with each one-standard-deviation (SD) increase in CTI. Analyses were performed using the fully adjusted multivariable logistic regression model (Model 3), adjusted for age, sex, body mass index, history of hypertension, history of diabetes, estimated glomerular filtration rate, history of surgery, and history of chemotherapy. This analysis was conducted as an exploratory assessment to evaluate the association between CTI and different cardiovascular event categories. CTI, C-reactive protein–triglyceride–glucose index.

**Supplementary Table 4. Performance Comparison of Machine Learning Models for Predicting Incident Cardiovascular Disease**

| **Model** | **AUC** | **Accuracy** | **Sensitivity** | **Specificity** | **Brier_Score** |
| --- | --- | --- | --- | --- | --- |
| **KNN** | 0.706 | 0.715 | 0.221 | 0.901 | 0.178 |
| **GLM** | 0.718 | 0.738 | 0.211 | 0.937 | 0.179 |
| **RF** | 0.684 | 0.744 | 0.189 | 0.952 | 0.181 |
| **SVM** | 0.670 | 0.755 | 0.179 | 0.972 | 0.177 |

Note: Performance metrics were evaluated in the primary Chinese cohort (n = 1,160) using fivefold cross-validation. Four machine learning algorithms were compared: K-nearest neighbors (KNN), generalized linear model (GLM), random forest (RF), and support vector machine (SVM).

**Supplementary Table 5. Sensitivity analysis of the association between CTI and incident cardiovascular disease after excluding participants with elevated baseline CRP levels**

| **Population** | **N** | **Events** | **OR per SD increase (95% CI)** | ***P*** |
| --- | --- | --- | --- | --- |
| Overall cohort | 1160 | 319 | 1.18 (1.02–1.36) | 0.025 |
| CRP ≤10 mg/L cohort | 699 | 178 | 1.29 (0.95–1.77) | 0.105 |

Note: Values represent odds ratios (ORs) and 95% confidence intervals (CIs) for incident cardiovascular disease associated with each one-standard-deviation (SD) increase in CTI. The sensitivity analysis was performed after excluding participants with elevated baseline CRP concentrations (>10 mg/L). All estimates were derived from the fully adjusted multivariable logistic regression model (Model 3), adjusting for age, sex, body mass index, history of hypertension, history of diabetes, estimated glomerular filtration rate, history of surgery, and history of chemotherapy. CTI, C-reactive protein–triglyceride–glucose index; CRP, C-reactive protein.

## 1.2 Supplementary Figures

**Supplementary Figure 1. Study Flow Diagram for Patient Selection in the UK Biobank Validation Cohort**

Note: Flow chart illustrating the selection process of rectal cancer patients from the UK Biobank database between January 2006 and August 2025. A total of 502,007 participants were initially recruited in the UK Biobank. Among these, 2,041 patients with an incident diagnosis of rectal cancer were identified using ICD-10 codes C19-C20. A total of 1,752 eligible rectal cancer survivors were included in the final validation cohort. During follow-up, 442 patients developed secondary cardiovascular disease (CVD), while 1,310 patients remained free of CVD. CRP, C-reactive protein; CTI, C-reactive protein-triglyceride-glucose index; CVD, cardiovascular disease; ICD-10, International Classification of Diseases, Tenth Revision.

**Supplementary Figure 2. Subgroup-Specific Dose-Response Relationships Between CTI and Incident Cardiovascular Disease**

Note: Restricted cubic spline analysis revealed the association between CTI and cardiovascular event risk in rectal cancer survivors, stratified by sex and age groups. CTI was modeled as a continuous variable, with the median CTI as the reference, and odds ratios were estimated at selected CTI values. Odds ratios are presented on a logarithmic scale to facilitate comparisons across subgroups.

**Supplementary Figure 3. LASSO Regression for Feature Selection and Penalty Parameter Tuning**

(A) LASSO cross-validation plot displaying the binomial deviance as a function of the log-transformed penalty parameter [log(λ)]. The two vertical dashed lines indicate the optimal values: the left line corresponds to λ_min (the value minimizing the mean cross-validated error), and the right line corresponds to λ_1se (the largest value within one standard error of λ_min, representing a more parsimonious model). The optimal λ was selected based on these criteria, balancing model fit and complexity.

(B) LASSO coefficient profiles showing the regularization paths for each candidate predictor variable as a function of log(λ). Each colored line represents the coefficient trajectory for a specific variable as the penalty parameter increases. As λ increases (moving from left to right on the x‑axis), the shrinkage penalty strengthens, and coefficients are progressively shrunk toward zero. Variables with coefficients that remain non‑zero at the selected λ value are retained in the final model.

**Supplementary Figure 4. Decision Curve Analysis and Calibration Plots for Models Incorporating CRP, TyG, and CTI**

(A) Decision curve analysis evaluating the clinical net benefit of three multivariable models for predicting incident cardiovascular disease (CVD) in rectal cancer survivors (n = 1,160). The x‑axis represents the threshold probability for predicting CVD, reflecting the relative weight assigned to false-positive versus false-negative predictions in clinical decision-making. The y‑axis represents the net benefit, calculated as the proportion of true positives minus the proportion of false positives weighted by the odds of the threshold probability.

Three prediction models were compared, all adjusted for the same covariates as in Model 3 of Table 2, with the addition of different inflammatory‑metabolic markers:

Model 1 (green line): C-reactive protein (CRP) alone

Model 2 (blue line): Triglyceride-glucose index (TyG) alone

Model 3 (red line): C-reactive protein-triglyceride-glucose index (CTI)

(B) Calibration curves assessing the agreement between predicted probabilities and observed outcomes for the three models. The x-axis represents the predicted probability of CVD from each model, and the y-axis represents the observed proportion of patients who developed CVD. The diagonal dashed line (y = x) indicates perfect calibration, where predicted probabilities exactly match observed outcomes. Points below the diagonal indicate overestimation of risk; points above the diagonal indicate underestimation.

All three models demonstrated good calibration, with no evidence of poor fit (all Hosmer–Lemeshow P > 0.05). The CTI model exhibited the best calibration, as evidenced by the highest P value and the lowest Brier score, indicating the closest agreement between predicted and observed probabilities.

Together, these analyses demonstrate that the CTI model not only provides superior discriminative ability (Table 3, Figure 3) and clinical net benefit but also maintains excellent calibration, supporting its reliability for individualized risk prediction in rectal cancer survivors.

CRP, C-reactive protein; TyG, triglyceride-glucose index; CTI, C-reactive protein-triglyceride-glucose index; CVD, cardiovascular disease.
